# Real-world challenges in hepatocellular carcinoma in Central America and the Caribbean: insights from a multinational expert survey

**DOI:** 10.3389/fonc.2025.1671564

**Published:** 2025-10-20

**Authors:** Christie Perelló, Hugo J. Disla, Pablo Coste, Marisabell Valdez, Dayron Páez, Carlos Rivera, Yong Loo, Tania Mayorga, Kenia Torres, Edwyn Pol, Enrique Adames, Miguel Mayo, Louis M. Morel, Rafael Gutierrez, José Luis Calleja

**Affiliations:** 1 Hepatology Unit, Digestive Disease Center, Hospital Metropolitano de Santiago (HOMS), Santiago, Dominican Republic; 2 Research Institute Puerta de Hierro Segovia de Arana (IDIPHISA), Madrid, Spain; 3 Liver Unit, Hospital Rafael Ángel Calderón Guardia, Liver Lab CR, San José, Costa Rica; 4 FACG, Clínica Médicos Especialistas, MEDIGASTRO Group, San Salvador, El Salvador; 5 Hospital Clínico Quirúrgico Hermanos Ameijeiras, Havana, Cuba; 6 Hospital Escuela and Honduras Medical Center, Tegucigalpa, Honduras; 7 Instituto Oncológico Nacional, Panama City, Panama; 8 Hospital Militar Escuela Dr. Alejandro Davila Bolaños, Managua, Nicaragua; 9 Hospital General Plaza de la Salud, Santo Domingo, Dominican Republic; 10 Centro Médico Humberto Molina, Oncology Medical Clinic, Quetzaltenango, Guatemala; 11 Department of Medicine, Universidad de Panamá & Gastroenterology Service, Hospital Santo Tomás, Panama City, Panama; 12 Internal Medicine and Gastroenterology Department, Universidad de Panamá, Panama City, Panama; 13 Department of Radiology, Centros de Diagnóstico y Medicina Avanzada y de Conferencias Médicas y Telemedicina (CEDIMAT), Santo Domingo, Dominican Republic; 14 Cancer Center, Hospital Metropolitano de Santiago (HOMS), Santiago, Dominican Republic; 15 Department of Gastroenterology and Hepatology, Hospital Universitario Puerta de Hierro, Universidad Autónoma Madrid, Madrid, Spain

**Keywords:** hepatocellular carcinoma, liver cancer epidemiology, cancer screening and surveillance, cancer diagnosis and imaging, liver cancer landscape, cancer epidemiology and prevention, immunotherapy in oncology

## Abstract

Hepatocellular carcinoma (HCC) represents a growing public health concern in Central America and the Caribbean, yet regional data on its management remain limited. A cross-sectional descriptive survey was conducted among 51 liver cancer specialists across nine countries, exploring four domains: epidemiology, screening, diagnostics, and treatment access. Analysis of the 20-item questionnaire revealed significant disparities in HCC care across the region. National cancer registries and structured screening programs were largely absent. Although ultrasound was widely available, its routine use for surveillance was inconsistent, and radiologist training levels varied. Access to diagnostic imaging such as triphasic computed tomography and magnetic resonance imaging was uneven. While liver surgery was generally accessible, liver transplantation was limited to Costa Rica and, to a lesser extent, the Dominican Republic. Systemic therapies such as sorafenib and lenvatinib were commonly available, However, access to first-line immunotherapy was constrained by limited insurance coverage and delayed governmental approval. In several countries, fewer than 30% of patients received treatment approval, with delays exceeding five months. Multidisciplinary tumor boards were not routinely implemented. In conclusion, these findings provide the first regional overview of HCC management in Central America and the Caribbean, underscoring critical gaps in surveillance infrastructure, diagnostic capacity, therapeutic access, and institutional coordination. Addressing these gaps is essential to improving liver cancer outcomes and equity across the region.

## Introduction

Hepatocellular carcinoma (HCC) is the most common form of primary liver cancer in adults and represents an increasing burden on healthcare systems worldwide (1).

HCC is an epithelial-origin tumor that typically emerges in the sixth to seventh decade of life and predominantly affects men, with a male-to-female ratio of 2:1. In recent decades, both the incidence and prevalence of HCC have been rising, making it the sixth most common cancer and the third leading cause of cancer-related death globally (2).

Although HCC has traditionally been associated with chronic liver damage mostly secondary to chronic infections by hepatotropic viruses, such as hepatitis B virus (HBV) and hepatitis C virus (HCV), its epidemiological profile is undergoing significant changes. Particularly in Western countries, liver diseases associated with metabolic dysfunction—such as metabolic dysfunction-associated steatotic liver disease (MASLD)—and alcohol abuse are becoming the leading risk factors, surpassing viral etiologies (3).

Given the clinical complexity of patients who often present with both cirrhosis and cancer, multidisciplinary management is essential. Accordingly, international guidelines emphasize the need for multidisciplinary teams in the management of HCC, comprising hepatologists, hepatobiliary and transplant surgeons, medical and radiation oncologists, radiologists, interventional radiologists, and specialized nursing staff. Increasingly, patients and their representatives should also be involved in the decision-making process, as they can contribute valuable and complementary perspectives (4).

**Figure 1:**
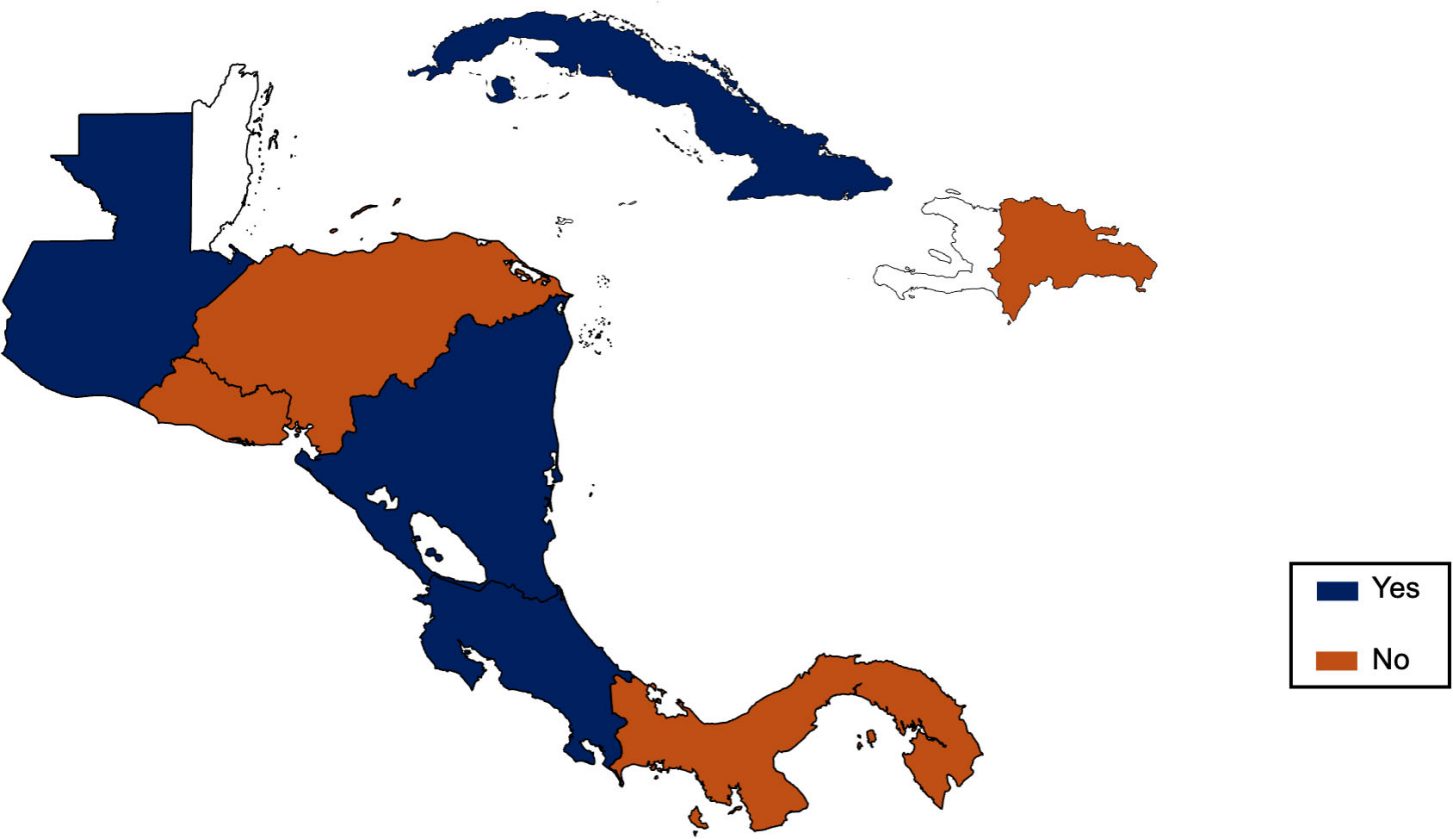
Epidemiological studies on hepatocellular carcinoma by Country in the Central America and Caribbean Region.

In Central America and the Caribbean, the management of HCC is heterogeneous and, in many regions, poorly understood. There is a notable scarcity of comprehensive population-based data, and existing information often derives from fragmented or institution-specific reports (**Figure 1**). Therefore, a meeting was organized to establish a multidisciplinary group of liver cancer experts—including hepatologists, oncologists, hepatic surgeons, and radiologists—coordinated from the Metropolitan Hospital of Santiago, Dominican Republic. Through a structured 20-item survey, the group sought to provide a critical and updated overview of the current state of HCC diagnosis and treatment across the region, with a focus on identifying major barriers, unmet needs, and opportunities for improvement.

This document aims to serve as a practical tool for healthcare professionals, public policy makers, and scientific organizations interested in improving the comprehensive management of liver cancer in the region.

## Materials and methods

### Survey design

A structured, self-administered online survey was developed by a multidisciplinary panel of experts in Liver Cancer to assess the current state of HCC care in Central America and the Caribbean. The 20-item questionnaire, administered in Spanish, as all participants were based in countries where Spanish is the official primary language, was designed to evaluate four main fields: epidemiology, screening strategies, diagnostic modalities, and therapeutic approaches. Prior to regional dissemination, the instrument underwent pilot testing with hepatocellular carcinoma specialists from the medical staff of the Hospital Metropolitano de Santiago to validate its clarity, relevance, and content integrity. The estimated time to complete the survey was approximately 8 minutes. The complete questionnaire is provided in **Supplementary Material 1**.

### Survey participation and data collection

The survey was distributed via email between May 8 and June 8, 2025, using a REDCap-hosted secure link. A purposive sampling strategy was employed to identify eligible participants, who were required to be physicians with demonstrated expertise in liver cancer management across the region. These experts have on average, a decade of experience managing patients with hepatocellular carcinoma and conduct their clinical practice within specialized services at referral hospitals in their respective countries. The most represented specialties were oncology (n=22) and gastroenterology (n = 19), followed by surgery (n = 8) and interventional radiology (n= 2).

Upon receipt of the participants’ responses, a second round of review was conducted to ensure the accuracy, completeness, and evidentiary support of the submitted data. This process also addressed any items left unanswered in the initial round. This follow-up was initiated via email correspondence, which also served to mitigate the risk of non-response bias. A full (100%) response rate was achieved. The preliminary results were then presented in a hybrid meeting, with both in-person and virtual attendance, enabling formal validation of the findings and resolution of any outstanding issues.

### Ethics statement

Given that this was an anonymous, minimal-risk study conducted among healthcare professionals, formal documentation of informed consent was not required. Completion and submission of the questionnaire were considered to imply consent. Participant anonymity was preserved throughout the process, and no identifiable personal information was collected. The study protocol was reviewed and deemed exempt from full ethics review in accordance with applicable regulations.

## Results

### Health system overview in Central America and the Caribbean

Understanding the structure and characteristics of national healthcare systems is essential to contextualize access barriers and care disparities across Central America and the Caribbean. Information regarding the characteristics of the healthcare systems in the participating countries was predefined and provided by each of the expert respondents. This ensured that the contextual descriptions were accurate and reflective of the national settings in which they practice.

A summary of the healthcare system structures in the participating countries is presented in **Table 1**.

**Table 1:** Comparative overview of health systems in Central America and the Caribbean.

Country	Health system type	Main public institutions	Private sector role	HCC treatment access
Costa Rica	Universal, solidarity-based (public-private mix)	CCSS	30% use for faster diagnostics and consultations	Available through public system
Cuba	Fully public and universal	Ministry of Public Health	Limited or non-existent	Organized via provincial tumor boards
Dominican Republic	Public-private	Ministry of Public Health, SNS, ARS SENASA	Covers Contributory Regime; copayments and exclusions apply	Limited coverage for advanced treatments like immunotherapy
El Salvador	Public-private	Integrated Health System, ISSS	10% coverage; faster, higher-quality care	Not specified
Guatemala	Public-private-NGO	MSPAS, IGSS	Urban-based, advanced and specialized services	Not specified
Honduras	Three subsystems: public, IHSS, private	Ministry of Health, IHSS	Out-of-pocket or insurance-based access	Not specified
Panama	Mixed model: public-private	CSS, Ministry of Health	10% insured; access to FDA/EMA therapies	Available at ION for all patients
Nicaragua	Mixed: public, INSS, private	MINSA, INSS	Faster, high-tech services; accessible to higher-income groups	Limited in public sector; better in private

This table summarizes the structural organization of national health systems and the reported access to hepatocellular carcinoma (HCC) treatments across eight countries in the region. It includes the health system type, main public institutions, the role of the private sector, and the availability of HCC-specific therapies.

CCSS, Caja Costarricense de Seguro Social; SNS, Servicio Nacional de Salud; ARS SENASA, Administradora de Riesgos de Salud del Estado; ISSS, Instituto Salvadoreño del Seguro Social; MSPAS, Ministerio de Salud Pública y Asistencia Social; IGSS, Instituto Guatemalteco de Seguridad Social; IHSS, Instituto Hondureño de Seguridad Social; CSS, Caja de Seguro Social; MINSA, Ministerio de Salud; INSS, Instituto Nicaragüense de Seguridad Social; ION, Instituto Oncológico Nacional; FDA, Food and Drug Administration; EMA, European Medicines Agency.

**Table 2:** Availability of hepatocellular carcinoma services across Central America and the Caribbean.

Country	HCC Registry	Structured screening	TAC access	Immunotherapy availability	Liver transplant availability	Multidisciplinary care
Cuba	Yes	No	Yes	Yes	No	Yes
Costa Rica	Yes	No	Yes	Yes	Yes	Yes
Dominican Republic	No	No	Yes	No	Partial	Occasional
El Salvador	No	No	Yes	Yes	No	Occasional
Guatemala	Yes	No	Yes	No	No	Yes
Honduras	No	No	Yes	No	No	Occasional
Nicaragua	Yes	No	Yes	Yes	No	Yes
Panamá	Yes	Yes	Yes	Yes	No	Occasional

This table summarizes the availability of HCC-related diagnostic and therapeutic services across eight countries. Variables include the existence of HCC registries, structured screening programs, access to CT imaging, immunotherapy availability, liver transplantation access, and the implementation of multidisciplinary care. “Partial” indicates limited or inconsistent availability. “Occasional” reflects non-structured or infrequent implementation of multidisciplinary case discussions.

**Cuba** has a public, free, and universal healthcare system organized into three levels: primary care (clinics and polyclinics), secondary care (general hospitals), and tertiary care (National Institutes and the Hermanos Ameijeiras Hospital). The primary and secondary levels are managed by provincial health directorates, while the tertiary level falls under the Ministry of Public Health. Multidisciplinary Tumor Boards for digestive tract malignancies, including HCC, are present in all provinces.

In contrast, **El Salvador’s** healthcare system is divided into public and private sectors. The public system includes MINSAL, which serves 70% of the uninsured population and operates the National Radiotherapy Center; the ISSS, covering 25% of formal workers and their families with an oncology hospital; as well as the Military Hospital and the Teachers’ Welfare Institute. The private sector, covering about 10% of the population, offers faster, higher-quality care, but is limited to those with private insurance or the ability to pay.

In **Honduras**, the healthcare system is segmented into three subsystems. The public subsystem, funded by the government and managed by the Ministry of Health, is organized into three levels: primary care, regional hospitals with specialized services, and high-tech hospitals for complex cases. The second subsystem is the Honduran Social Security Institute (IHSS), which provides care to formal workers and their families. The third is the private sector, consisting of clinics, hospitals, and laboratories offering services to individuals who can pay directly or through private insurance.

**Panama** presents a mixed model composed of a public sector—represented by the Social Security Fund (CSS) and the Ministry of Health—and a private sector. Public oncology care is centralized at the National Oncology Institute (ION), which serves both insured and uninsured patients. Approximately 10% of the population has private health insurance that covers 70% to 100% of oncology-related costs and allows access to all FDA- or EMA-approved therapies.

In **Nicaragua**, the system is also mixed, including a public sector, the Nicaraguan Social Security Institute (INSS), and a private sector. The Ministry of Health (MINSA) is the primary provider, offering free, universal care—especially to low-income populations—through a network of hospitals, health centers, and clinics. However, the system faces challenges such as staffing shortages and limited resources, particularly in rural areas. The INSS provides healthcare coverage to formal workers and their dependents via contracted private clinics and hospitals, but it does not extend to informal workers or the unemployed. The private sector offers faster service and better technology, though at a higher cost, accessible mainly to higher-income individuals or those with private insurance.

**Costa Rica** is known for a solidarity-based and universal healthcare system led by the Costa Rican Social Security Fund (CCSS), financed by the state, employers, and workers. The CCSS covers about 90% of the population, offering comprehensive services ranging from primary care to specialized treatments and medication supply. A growing private sector serves 30% of the population, offering quicker consultations and advanced diagnostics.

In the **Dominican Republic**, the healthcare system is divided into public and private sectors. The public one is overseen by the Ministry of Public Health and managed by the National Health Service (SNS), which operates the hospital network and provides care to vulnerable populations. It is financed by the state and administered by the public Health Risk Administrator (ARS SENASA). While this system covers some catastrophic illnesses, it has limitations regarding advanced oncologic treatments for HCC, including immunotherapies not listed in the basic benefits package. The private sector, accessed via contributory insurance schemes, often requires significant co-payments or additional authorization for innovative treatments.

Lastly, in **Guatemala**, the healthcare system consists of public, private, and non-governmental sectors. The Ministry of Public Health and Social Assistance (MSPAS) provides free public services, particularly in rural areas. The Guatemalan Social Security Institute (IGSS) supports formal workers and their families, while the private sector, concentrated in urban regions, offers specialized services at higher costs, accessible mainly to those with insurance or the ability to self-finance care.

### Epidemiology and disease burden (Questions 1 – 3)

Epidemiological studies on HCC in Central America and the Caribbean are limited and unevenly distributed. Costa Rica and Nicaragua reported four national studies on HCC, while Cuba cited two and El Salvador, Guatemala, and the Dominican Republic one. A noteworthy study from Costa Rica found that only 43.5% of HCC cases were detected through screening, with an average delay of 70 days to confirm diagnosis. Additionally, a 90.5% increase in incidence was observed during the study period, highlighting significant gaps in early detection (5). Structured epidemiological research is largely lacking, with most studies limited to selected cohorts and limited national generalizability.

Epidemiological research on liver disease in the region is limited, despite a high disease burden. Alcohol-related liver disease predominates in the Dominican Republic and El Salvador, while metabolic causes are more common in Costa Rica, Nicaragua, and Guatemala. Viral causes are less frequent. Strengthening surveillance and prioritizing population-based studies is urgently needed.

The availability of liver cancer mortality data varies across the region, with consistent reporting in Cuba, Guatemala, and Nicaragua, and updated registries in Costa Rica. In 2023, Cuba recorded 798 deaths from malignant liver and intrahepatic bile duct tumors (7.2 per 100,000), predominantly among men (65%) (6).

In contrast, Honduras, El Salvador, and the Dominican Republic face major limitations. In the Dominican Republic, although data are available from oncology hospitals, no centralized national registry exists. Panama has a national registry but does not distinguish between HCC and cholangiocarcinoma, grouping both under the term liver cancer. Disparities in the availability and reliability of data present a substantial barrier to the design of evidence-based public health strategies in the region.

The convergence of limited research capacity and a high burden of HCC risk factors highlights the urgent need to strengthen regional cancer registries for both incidence and mortality. Promoting population-based studies is essential to generate accurate and representative epidemiological data.

### Screening and surveillance (Questions 4 – 7)

While ultrasound is perceived as widely available for HCC screening in all evaluated countries, its effective implementation faces key limitations. Most countries lack structured screening programs, limiting systematic use and impact on early detection. Data on the proportion of cirrhotic patients receiving screening ultrasound are generally unavailable, with some limited reports from Cuba and Costa Rica. Panama has endorsed a National Consensus for HCC Screening, Diagnosis, and Management, which defines a structured surveillance approach based on biannual ultrasound and alpha-fetoprotein testing (7).

Survey responses indicate that in most Central American and Caribbean countries, health insurance is not perceived as a major barrier to conducting biannual ultrasounds in patients with cirrhosis. Beyond financial considerations, the main barriers identified for HCC screening were the failure to recognize eligible patients and the low frequency with which specialists request ultrasound screenings. These findings highlight the need to raise awareness among healthcare professionals, and possibly patients, regarding the importance of HCC screening in individuals with advanced liver disease.

The adequacy of ultrasound training for HCC screening varies across countries. In both the Dominican Republic and El Salvador, radiologists are generally considered well-trained. However, many ultrasound examinations are performed by physicians who are not formally trained as radiologists. Respondents in Honduras and Nicaragua reported inadequate training, while feedback from Costa Rica was mixed. Concerns in Cuba focused on non-standardized reporting, and responses from Guatemala and Panama were largely negative. Overall, there is a regional need to improve and standardize ultrasound training.

The frequency of biannual AFP testing requests for cirrhotic patients varies considerably across the region. In the Dominican Republic, Costa Rica, El Salvador, Cuba, Panama, and Guatemala, the practice appears relatively common as part of HCC screening. In Honduras and Nicaragua, AFP testing is less commonly used, possibly due to limited availability or low guideline adherence. Despite some flaws and pitfalls, the role of specific biomarkers as alternative or complementary diagnostic tools for the current standard of care for early-stage diagnosis of HCC is being intensively researched in this context (8). The utilization of serological biomarkers, such as des-γ-carboxy prothrombin (DCP), has the potential to enhance screening and surveillance practices in the region.

### Diagnostic techniques and radiologic criteria (Questions 8 – 11)

Access to computed tomography (CT) and magnetic resonance imaging (MRI) varies considerably across the region. CT is more widely available, particularly in the Dominican Republic, Costa Rica, and Honduras. However, MRI access is more limited and uneven—even in countries with broad CT coverage. While CT is relatively accessible, the restricted availability of MRI represents a challenge for the timely and accurate diagnosis of both benign and malignant liver lesions.

Insurance coverage for contrast-enhanced triple-phase CT varies across the region. In the Dominican Republic, coverage is often partial, limiting full imaging. El Salvador, Costa Rica, Nicaragua, Guatemala, and Panama generally offer full coverage, while Cuba and Honduras have significant restrictions. These inconsistencies may hinder timely and equitable HCC diagnosis.

Most physicians reported confidence in the ability of radiologists in their respective countries to diagnose HCC using international non-invasive criteria. Despite some national variation, the overall perception of radiological expertise in this area was positive across the region.

The requirement for biopsy to approve treatment varied across countries. While biopsy is not mandatory in most settings, physicians in the Dominican Republic, El Salvador, Honduras, and Nicaragua reported that it is still commonly required. These differences likely reflect variations in national regulations, institutional protocols, or access to diagnostic technology.

### Treatment modalities (Questions 12 – 13)

Availability of ablative therapies for HCC varied markedly across the region. The Dominican Republic, Panama, Costa Rica, and El Salvador reported the broadest access, including radiofrequency and microwave ablation, alcohol injection, stereotactic body radiotherapy (SBRT), and chemoembolization. In contrast, Guatemala and Nicaragua, reported more limited access, while Cuba primarily relied on alcohol injection and chemoembolization. These findings underscore substantial disparities in therapeutic resources, with potential implications for clinical outcomes and the standardization of care.

In terms of surgical management, most countries reported sustained access to specialized liver surgery. Perceptions in El Salvador were mixed, possibly reflecting geographic disparities in availability.

Access to liver transplantation remains limited across the region. Costa Rica was the only country to report consistent availability, while the Dominican Republic and Panama noted partial access with significant constraints. Reported barriers included low organ donation rates, limited insurance coverage, and high out-of-pocket expenses. No access to liver transplantation was reported in Cuba, El Salvador, Guatemala, Honduras, or Nicaragua.

### Access to systemic therapy (Questions 14 – 17)

Systemic therapies for HCC were evaluated, including tyrosine kinase inhibitors (TKIs) and immunotherapy combinations. Sorafenib and Lenvatinib were the most widely reported drugs, especially in the Dominican Republic, Panama, Honduras, Nicaragua, El Salvador, and Costa Rica. The combination of Atezolizumab + Bevacizumab was also available, particularly in the Dominican Republic, Panama and Costa Rica. The combination of Durvalumab + Tremelimumab was reported only in isolated cases. These findings reveal uneven access to systemic therapies, with persistent gaps in the availability of newer or combined regimens, limiting access to evidence-based treatments across the region.

Significant disparities in insurance coverage for immunotherapy were identified across Central America and the Caribbean. Respondents from Costa Rica, El Salvador, Panama, Nicaragua, and Cuba reported predominantly positive coverage, while those from Honduras, Guatemala, and the Dominican Republic indicated no coverage. These findings highlight a key structural barrier to equitable access to cancer immunotherapies in the region.

Government authorization was commonly reported as a regulatory barrier to timely HCC treatment. In the Dominican Republic, Nicaragua, Honduras, Cuba, and Costa Rica, most physicians indicated that prior approval is required. In contrast, all respondents in Panama reported no such requirement. Responses from El Salvador were mixed. In the Dominican Republic, prescriptions must be issued by an oncology specialist, regardless of their experience with liver tumors, and are systematically rejected if written by hepatologists or other specialists trained in managing liver cancer patients.

Significant variation exists in both approval rates and authorization times. In the Dominican Republic and El Salvador, fewer than 30% of patients receive approval, with delays ranging from one to over five months raising the question of treatment futility. Costa Rica showed a more favorable scenario, with higher approval rates (between 50% and >70%) and shorter delays (one to three months). Cuba reported high approval with an estimated one-month delay, and Panama reported no delays.

These findings collectively highlight stark regional disparities in access to systemic therapies, shaped by drug availability, insurance coverage, and regulatory processes. These structural gaps may significantly affect the timeliness and equity of care, ultimately influencing survival outcomes in patients with advanced HCC.

### Real-world use of HCC therapies (Questions 18–19)

Use of systemic therapies for HCC in real-world settings across Central America and the Caribbean reveals pronounced inter-country variability, influenced by differences in access, healthcare infrastructure, and clinical practice patterns.

Regarding of Tirosin Kinase Inhibitors (TKIs), the number of HCC patients treated within the last year varied substantially among respondents. In the Dominican Republic, most physicians reported treating between zero and three patients, suggesting limited utilization despite the availability of these agents. Physicians noted that TKIs are typically used when immunotherapy is unavailable and that their approval process is generally simpler and cheaper. A similar pattern was observed in El Salvador, Cuba, Panama, and Honduras.

Use of immunotherapy varied widely. The Dominican Republic had the highest number of physicians reporting no immunotherapy use (n = 14), followed by El Salvador (n = 8) and Honduras (n = 4). In these countries, as well as Guatemala, limited use may be due in part to the advanced clinical stage at diagnosis, which often precludes immunotherapy due to safety concerns or therapeutic futility. In contrast, Panama, Costa Rica, Nicaragua, and Cuba showed greater availability or integration of immunotherapy into clinical practice.

These findings underscore regional disparities in both access to and clinical implementation of immunotherapy. Variability is influenced by factors such as disease stage at presentation, insurance coverage, and national health policies, all of which may impairs treatment outcomes for patients with HCC.

### Multidisciplinary care (Question 20)

The implementation of multidisciplinary tumor boards for HCC varies widely across the region. While countries like Cuba, Costa Rica, and Guatemala reported regular meetings, others—including El Salvador, Honduras, Panama, and the Dominican Republic—indicated infrequent use. This limited and uneven integration may impact the quality and consistency of treatment decisions.

## Limitations

The conclusions of this survey should be interpreted with caution, as the limited number of professionals surveyed may not fully reflect the diversity of practices and realities across the region. In addition, the uneven geographical distribution of respondents and the inherent constraints of a cross-sectional design may limit the generalizability of the findings.

## Conclusions

Central America and the Caribbean face numerous structural and equity-related challenges in the management of hepatocellular carcinoma (HCC). Persistent gaps exist in epidemiological research, diagnostic access, therapeutic availability (including immunotherapy and liver transplantation), and the institutionalization of multidisciplinary teams. Such disparities compromise early detection and timely access to treatment, with potential negative consequences for patient prognosis. **Table 2** summarizes this information providing an overview of the structural and equity-related challenges that affect research, diagnosis, treatment access, and multidisciplinary management of HCC in the region.

Based on this survey, the following recommendations are proposed:

1. Promote the development of national epidemiological registries to obtain reliable data on HCC incidence, mortality, and risk factors.
2. Establish national screening programs using ultrasound and alpha-fetoprotein every 6 months in cirrhotic patients, following international guidelines.
3. Strengthen training for radiologists and treating physicians in imaging-based and non-invasive diagnostic criteria for HCC.
4. Develop targeted training programs for all specialists involved in the clinical management of HCC.
5. Expand access to innovative therapies, particularly systemic immunotherapy, by incorporating them into national formularies and insurance coverage, ensuring streamlined and effective prescription.
6. Remove barriers to access to treatment expanding the spectrum of prescribing physicians, including hepatologist and other specialist with dedication to liver cancer.
7. Promote the formation and institutionalization of multidisciplinary teams at all levels of hospital care.
8. Encourage public and professional awareness campaigns on the importance of early diagnosis.
9. Given the geographic proximity of the countries in this region, the establishment of a strategic alliance may represent an effective approach to overcoming the barriers to hepatocellular carcinoma management in this area. A regional framework could strengthen data collection and research initiatives, fostering evidence-based strategies tailored to the specific epidemiological context of the region.

## Data Availability

The datasets presented in this article are not readily available. The raw, anonymized dataset generated for this study is available from the corresponding author upon reasonable request. Access is restricted to protect participant confidentiality, in accordance with the terms of the informed consent and the study’s ethical approval.
