# Correction: Real-world challenges in hepatocellular carcinoma in Central America and the Caribbean: insights from a multinational expert survey

**DOI:** 10.3389/fonc.2025.1728925

**Published:** 2025-11-13

**Authors:** Christie Perelló, Hugo Disla, Pablo Coste, Marisabell Valdez, Dayron Páez, Carlos Rivera, Yong Loo, Tania Mayorga, Kenia Torres, Edwyn Pol, Enrique Adames, Miguel Mayo, Louis Morel, Rafael Gutierrez, José Luis Calleja

**Affiliations:** 1 Hepatology Unit, Digestive Disease Center, Hospital Metropolitano de Santiago (HOMS), Santiago, Dominican Republic; 2 Research Institute Puerta de Hierro Segovia de Arana (IDIPHISA), Madrid, Spain; 3 Liver Unit, Hospital Rafael Ángel Calderón Guardia, Liver Lab CR, San José, Costa Rica; 4 FACG, Clínica Médicos Especialistas, MEDIGASTRO Group, San Salvador, El Salvador; 5 Hospital Clínico Quirúrgico Hermanos Ameijeiras, Havana, Cuba; 6 Hospital Escuela and Honduras Medical Center , Tegucigalpa, Honduras; 7 Instituto Oncológico Nacional , Panama City, Panama; 8 Hospital Militar Escuela Dr. Alejandro Davila Bolaños, Managua, Nicaragua; 9 Hospital General Plaza de la Salud, Santo Domingo, Dominican Republic; 10 Centro Médico Humberto Molina, Oncology Medical Clinic, Quetzaltenango, Guatemala; 11 Department of Medicine, Universidad de Panamá & Gastroenterology Service, Hospital Santo Tomás, Panama City, Panama; 12 Internal Medicine and Gastroenterology Department, Universidad de Panamá, Panama City, Panama; 13 Department of Radiology, Centros de Diagnóstico y Medicina Avanzada y de Conferencias Médicas y Telemedicina (CEDIMAT), Santo Domingo, Dominican Republic; 14 Cancer Center, Hospital Metropolitano de Santiago (HOMS), Santiago, Dominican Republic; 15 Department of Gastroenterology and Hepatology, Hospital Universitario Puerta de Hierro, Universidad Autónoma Madrid, Madrid, Spain

**Keywords:** hepatocellular carcinoma, liver cancer epidemiology, cancer screening and surveillance, cancer diagnosis and imaging, liver cancer landscape, cancer epidemiology and prevention, immunotherapy in oncology

The affiliation for “Dr. José Luis Calleja” was erroneously given as

“2 Research Institute Puerta de Hierro Segovia de Arana (IDIPHISA), Madrid, Spain”,

“3 Liver Unit, Hospital Rafael Ángel Calderón Guardia, Liver Lab CR, San José, Costa Rica”,

“4 FACG, Clínica Médicos Especialistas, MEDIGASTRO Group, San Salvador, El Salvador”,

“5 Hospital Clínico Quirúrgico Hermanos Ameijeiras, Havana, Cuba”,

“6 Hospital Escuela and Honduras Medical Center, Tegucigalpa, Honduras”,

“7 Instituto Oncológico Nacional, Panama City, Panama”,

“8 Hospital Militar Escuela Dr. Alejandro Dávila Bolaños, Managua, Nicaragua”,

“9 Hospital General Plaza de la Salud, Santo Domingo, Dominican Republic”,

“10 Centro Médico Humberto Molina, Oncology Medical Clinic, Quetzaltenango, Guatemala”,

“11 Department of Medicine, Universidad de Panamá & Gastroenterology Service, Hospital Santo Tomás, Panama City, Panama”,

“12 Internal Medicine and Gastroenterology Department, Universidad de Panamá, Panama City, Panama”,

“13 Department of Radiology, Centros de Diagnóstico y Medicina Avanzada y de Conferencias Médicas y Telemedicina (CEDIMAT), Santo Domingo, República Dominicana”,

“14 Cancer Center, Hospital Metropolitano de Santiago (HOMS), Santiago, Dominican Republic”,

“15 Department of Gastroenterology and Hepatology, Hospital Universitario Puerta de Hierro, Universidad Autónoma Madrid, Madrid, Spain.”

The correct affiliation is:

“2 Research Institute Puerta de Hierro Segovia de Arana (IDIPHISA), Madrid, Spain” and

“15 Department of Gastroenterology and Hepatology, Hospital Universitario Puerta de Hierro, Universidad Autónoma Madrid, Madrid, Spain.”

The original version of this article has been updated.

