# Supplementary material for: Real-world challenges in hepatocellular carcinoma in Central America and the Caribbean: insights from a multinational expert survey
Source: Front Oncol. 2025 Oct 20;15:1671564. doi: 10.3389/fonc.2025.1671564 (PMC12580115; doi:10.3389/fonc.2025.1671564)
Supplement: Supplementary file 1 [file DataSheet1.pdf]

HCC - Central America

Thank you for participating in this survey on the current status of hepatocellular carcinoma (HCC) epidemiology, screening, diagnosis, and treatment in Central America. Your responses will help us better understand regional practices, existing challenges, and opportunities for improvement. Participation is voluntary, and all responses will be kept confidential and used exclusively for research purposes. Please answer each question based on your experience and knowledge. The survey will take approximately 10 to 15 minutes to complete. There are no right or wrong answers; we value your honest and professional input. Thank you very much for your valuable collaboration!

Name ______________________________

Email ______________________________

Specialty
- ◯ Gastroenterology
- ◯ Oncology
- ◯ Surgery

Country
- ◯ Costa Rica
- ◯ El Salvador
- ◯ Guatemala
- ◯ Honduras
- ◯ Nicaragua
- ◯ Panama
- ◯ Cuba
- ◯ Dominican Republic

Epidemiology

Are there specific epidemiological studies on liver cancer in your country?
- ◯ Yes
- ◯ No

Please elaborate on your answer: ______________________________

Are there epidemiological studies on liver disease in general in your country?
- ◯ Yes
- ◯ No

Please elaborate on your answer: ______________________________

What is the most common cause of disease in your country?
- ◯ Hepatitis C virus
- ◯ Hepatitis B virus
- ◯ Alcohol
- ◯ Metabolic Liver Disease
- ◯ Others

Are there mortality data related to cancer in your country?
- ◯ Yes
- ◯ No

Please elaborate on your answer: ______________________________

Are there mortality data related to mortality liver cancer in your country?
- ◯ Yes
- ◯ No

Please elaborate on your answer:

Screening

Is ultrasound generally available for screening in patients with liver disease?	◯ Yes<br>◯ No
Are insurance policies a barrier to performing two ultrasounds per year in patients with cirrhosis?	◯ Yes<br>◯ No
Do you believe radiologists in your country are adequately trained in ultrasound for HCC screening?	◯ Yes<br>◯ No
Are there national data available on the percentage of patients with cirrhosis who receive regular screening?	◯ Yes<br>◯ No
Do you routinely request alpha-fetoprotein tests every six months in your patients?	◯ Yes<br>◯ No
What do you consider the main barrier to HCC screening in your setting?	☐ Lack of patient identification<br>☐ Specialists do not request ultrasound<br>☐ Ultrasound not covered by insurance

Diagnosis

Is there access to computed tomography (CT) scans in most cities hospitals in your country?	◯ Yes<br>◯ No
Is there access to resonance magnetic (RM) scans in most cities hospitals in your country?	◯ Yes<br>◯ No
In case of clinical or ultrasound suspicion of liver cancer, does insurance cover a contrast-enhanced triphasic CT scan?	◯ Yes<br>◯ No
Are radiologist in your country adequately trained have to diagnose HCC using non-invasive international criteria?	◯ Yes<br>◯ No
Is biopsy required in your country to confirm the diagnosis and obtain approval for HCC treatment?	◯ Yes<br>◯ No

Treatment

What HCC ablative treatment techniques are available in your country?	☐ a. Radiofrequency ablation<br>☐ b. Microwave ablation<br>☐ c. Alcohol ablation<br>☐ d. SBRT (stereotactic body radiation therapy)<br>☐ e. Chemoembolization<br>☐ f. Radioembolization

Are there liver surgeons specialized in hepatic resection in your country?	◯ Yes<br>◯ No
Is liver transplantation available in your country?	◯ Yes<br>◯ No
Is it possible to refer patients to others countries for liver transplant?	◯ Yes<br>◯ No
Please elaborate on your answer:	__________________________________

Are the following systemic treatments available in your country?

	Yes	No
Sorafenib	◯	◯
Lenvatinib	◯	◯
Atezolizumab + Bevacizumab	◯	◯
Durvalumab / Tremelimumab	◯	◯

Is immunotherapy covered by insurance?	◯ Yes<br>◯ No
Please elaborate on your answer:	__________________________________
Does systemic treatment require centralized governmental approval in your country?	◯ Yes<br>◯ No
Please elaborate on your answer:	__________________________________
Are HCC cases discussed in a multidisciplinary tumor committee as part of the clinical decision-making process?	◯ Yes<br>◯ No<br>◯ Sometimes
Please elaborate on your answer:	__________________________________
What percentage of your patients receive approval for the requested systemic treatment?	◯ a. Less than 10%<br>◯ b. 10-30 %<br>◯ c. 30-50 %<br>◯ d. 50-70 %<br>◯ e. More than 70%
What is the estimated approval delay?	◯ a. 1 month<br>◯ b. 1-3 months<br>◯ c. 3-5 months<br>◯ d. More than 5 months

Approximately how many HCC patients were treated with tyrosine kinase inhibitors (TKIs) at your center in the past year?	◯ a. None<br>◯ b. 1-3<br>◯ c. 4-7<br>◯ d. 8-12<br>◯ e. More than 12
Approximately how many HCC patients were treated with immunotherapy at your center in the past year?	◯ a. None<br>◯ b. 1-3<br>◯ c. 4-7<br>◯ d. 8-12<br>◯ e. More than 12
